# A new experimental approach to manipulate social identity and social norms about protective behaviors in a virtual social environment

**DOI:** 10.1186/s40359-026-04591-6

**Published:** 2026-04-28

**Authors:** Sophie Kittelberger, Alexandra M. Freund, Urte Scholz

**Affiliations:** Department of Psychology, University of Zurich, Binzmuehlestr. 14, Box 11, Zurich, Switzerland

**Keywords:** Social identity, Social norms, Health-protective behaviors, Virtual social environment, Citizen Science

## Abstract

**Background:**

Focus theory of normative conduct and social identity theory propose that the degree of social identification with a group moderates the influence of social norms on behavior. The current study protocal reports two studies testing this causal assumption in the context of health protective behaviors during a health crisis using a newly developed experimental manipulation of both social identity and social norms regarding protective behaviors during a health crisis in a virtual social environment.

**Methods:**

A new virtual social environment was co-created with Citizen Scientists and will be used in two experiments covering several online sessions to test (a) the manipulation of social identity, and (b) the manipulation of social norms regarding protective behaviors in a simulated health crisis (Study 1: using a vignette approach; Study 2: using the virtual social environment). The main outcome is adherence to protective behaviors (Study 1: self-reported; Study 2: behavior within the virtual social environment).

**Results:**

We successfully developed and implemented the virtual social environment using a Citizen Scientist approach. The Citizen Scientists report high overall participation experiences co-creating the virtual social environment, as well as high learning gains in coordinating with others, using GitHub/Git, and game mechanics.

**Discussion:**

Both studies will provide insights into (1) the development of a virtual social environment in collaboration with Citizen Scientists, (2) methods for experimentally manipulating social identity and social norms, and (3) the causal mechanisms linking social identity, and social norms to protective behaviors. We expect that the findings inform methodological approaches for experimentally simulating social situations that typically lay outside experimental control (here: a health crisis) and assessing health-related behaviors, future Citizen Science-based approaches, and theory on social influence on behavior during a health crisis.

**Trial registration:**

The trial registration ID is ISRCTN97703902. We registered the studies with ISRCTN on February 6, 2025, with the last update on March 5, 2025.

During a health crisis such as the Covid-19 pandemic, adherence to government mandated protective health behaviors (henceforth called: protective behaviors) is central for limiting contagion [1–3]. Testing the causal role of theoretical constructs such as social identity with family or friendship groups and social norms on adherence to protective behaviors during such a health crisis requires an experimental approach. Given that it is impossible to experimentally induce a health crisis in real life, social norms, or a social identity with family or friendship groups, we developed a virtual social environment (VSE) to do so. We co-created this VSE together with Citizen Scientists and assess the protective behaviors of the player within the game (Study 2) and using a vignette (Study 1). This newly developed game will be tested in two pre-registered experiments [4, 5].

## Adopting protective behaviors during a health crisis

One of the central challenges to reduce the risk of infection during the Covid-19 pandemic was to motivate people to adopt effective protective behaviors such as wearing face masks, keeping social distance, regularly washing hands, and getting vaccinated [6]. Despite the effectiveness of these behaviors, a substantial percentage of people did not adhere to them: A multi-country survey conducted between June and December 2020 found that 32% of respondents did not adopt social distancing, 25.1% did not wear face masks, and 26.9% did not wash or sanitize their hands [7]. Health crises involving an infectious disease have a pronounced social component as contagion occurs between people; hence protection against contagion is not only an individual decision but has consequences for the immediate social group and, ultimately, the spread to society at large [8].

Why did people differ so widely in their adherence to protective behaviors? A theory-based approach is essential to understand and ultimately improve adherence to protective behaviors [9]. Social identity theory (SIT) [10, 11] and the focus theory of normative conduct (FTNC) [12, 13] suggest that one of the factors contributing to the adoption of protective behaviors are social norms of a group with whom one identifies. Particularly in times of uncertainty such as a pandemic, people may look for guidance by considering what others do and what they approve or disapprove of [6, 14]. In other words: Social norms and social identity likely play an important role in understanding adherence to protective behaviors [6]. Although there is longitudinal evidence that the norms of a social group with which one identifies are a reliable predictor of behavior [15–17], there is a lack of experimental evidence in the context of health crises. The reasons for this gap in the literature is that it is not possible to experimentally induce a health crisis, social identity, and social norms to test their causal influence on adherence to protective behaviors. To address this we developed a new experimental paradigm allowing to simulate a health crisis of pandemic proportions, to induce a social identity with a new social group, and to induce different social norms using a computer game (aka virtual social environment, VSE).

### Social norms

Social norms can be defined as socially shared expectations about appropriate behavior, and deviations from these expectations incur social sanctions ranging from legal consequences to social disapproval. Social norms may be injunctive (i.e., what ought to be done) or descriptive (i.e., what most people within a social group do) [12, 13].

Social norms strongly influence individual behavior in general [12], including health behaviors [18]. During a health crisis, protective behaviors introduced as government mandates are explicit and legally codified injunctive norms [19] (e.g., wearing a face mask in public transportation during the Covid-19 pandemic). Descriptive norms refer to perceptions of how prevalent certain behaviors are in a given social group (e.g., beliefs about how frequently others get vaccinated). Injunctive norms motivate action through anticipated social rewards or punishments [13]. Although injunctive and descriptive norms are often aligned, the Covid-19 pandemic demonstrated that they may also diverge [19]. The FTNC [12, 13] posits that injunctive and descriptive norms both independently and jointly affect behavior. A key dimension regarding the impact of social norms on behavior is the degree to which they are salient, i.e., the degree to which a social norm is more or less prevalent in a given situation. There is ample empirical evidence that both descriptive and injunctive norms influenced adherence to protective behaviors during the Covid-19 pandemic [19–27]. Other theoretical models such as the norm activation model (NAM; [28, 29]) state that people engage in prosocial behaviors (e.g., adhering to protective behaviors) because of moral obligation. According to NAM, the awareness of negative consequences of a threat such as the Coronavirus, the perceived personal responsibility to avoid a threat, and the personal norm of how to act in such a situation impact individual behavior. In fact, there is empirical evidence in the context of the Covid-19 pandemic supporting these assumptions of NAM [30, 31]. However, as is true for the bulk of research in the context of a health crisis, these studies are correlative and do not allow to draw causal inferences. For a stronger test of causal assumptions as those put forth by NAM, experimental approaches are essential.

### Social identity

Social identity denotes the degree to which people feel they belong to specific social groups, and include the evaluative and affective relationship with the groups [32, 33]. According to social identity theory (SIT) and self-categorization theory (SCT) [11, 34], the social identities of a person are part of their self-concept. In order to achieve and maintain a favorable self-concept, people strive to belong to positively evaluated social groups. Such a positive evaluation is derived through comparisons of one’s in-groups with out-groups [10, 34]. According to SIT, people achieve belonging to a positively evaluated in-group either by leaving a less positively evaluated in-group and joining a more positively evaluated group, or by making their current in-group more positively distinct [10, 34].

Several studies show that identifying with various social groups (e.g., family, friends, people living in one’s country) was related to the adherence to protective behaviors during the Covid-19 pandemic [22, 35, 36]. These findings were interpreted as reflecting a higher motivation to protect one’s in-group when strongly identifying with it [35]. Surprisingly, however, people also adhered less to protective behaviors when with close family or friends even when at risk of contagion, supposedly because they experienced lower disgust and risk when strongly identifying with the group [37–40]. To explain these seemingly contradictory results, we propose that the in-group’s social norms about protective behaviors moderate which behavior people are more likely to show. Social groups such as family and friendship groups often establish their own social norms regarding protective behaviors [6], and the higher the social identity with this group, the stronger should be the influence of these social norms on people’s behavior [10, 11, 34, 41].

### Virtual social environments as a psychological assessment tool

Virtual social environments (VSEs), implemented as computer games, offer a way to simulate a health crisis and experimentally manipulate social identity and social norms. VSEs represent a methodological middle ground between the high control of laboratory experiments and the external validity of observational studies in people’s everyday lives [42], making them promising tools for studies involving constructs that cannot be easily experimentally manipulated otherwise [43]. In a VSE, programmed (i.e., non-playable) avatars can be used to simulate dyadic or group interactions, and may represent different social groups (e.g., family and friendship groups). Compared to vignettes, VSEs offer a higher degree of immersion allowing VSEs to simulate complex social situations more effectively than static scenarios. Notably, immersion does not necessarily depend on the realism of the VSE: Symbolic or simplified representations of people can elicit similar psychological effects as realistic depictions, suggesting that high-end visual or technical requirements are not essential to induce an effective experimental context [42]. Since participants actively control the behavior of their avatar in the VSE, this approach also allows to collect behavioral data instead of, or in addition to, self-report measures that are more prone to biases than behaviors [44]. To complement behavioral assessments and tap into non-behavioral constructs such as emotional experiences, VSEs allow to include self-report measures. Note also that VSEs can be combined with vignette-based approaches, allowing researchers to simulate a social scenario with experimentally manipulated variables and subsequently present a vignette to assess, for instance, evaluations of alternative scenarios.

Despite these advantages, VSEs remain underutilized in psychological research, probably due to the complexity of developing even simple VSEs [42]. The available extant studies underscore the potential of VSEs as they demonstrate their successful application in the investigatation of social processes, including bystander effects [45], the impact of perceived physical height on speech anxiety during virtual job interviews [46], and interactions with a virtual spouse [42]. Particularly relevant in the present context, a study found that collaborative activities within the VSE „Second Life “ increased participants’ degree of social identification with the group [47]. This underscores the potential of VSEs for studying social identity and social identification processes. Due to licensing restrictions, we did not use „Second Life “ but co-created a novel VSE collaboratively with Citizen Scientists.

### Citizen Science

Over the past decades, social scientists have increasingly included the experience and knowledge of citizens into their research [48]. The framework of Public Participation in Scientific Research (PPSR) is one of various other forms of citizen participation in scientific research and defines it as „[…] intentional collaborations in which members of the public engage in the process of research to generate new science-based knowledge “ [49]. Citizen Science refers to citizen participation in research ranging from contributing to data collection to co-creating research, defined as „[…] develop a study and work with input from scientists to address a question of interest or an issue of concern “ [49, 50]. The advantages of Citizen Science co-creation include enhanced external validity, broader data reach, and improved public trust and engagement. Practical challenges include maintaining data quality, ethical complexities around consent and power-sharing, and the need for clear agreements on data ownership and intellectual property [49]. As will be described in more detail below, we involved Citizen Scientists in the co-creation of the computer game we used as a VSE for our studies. In fact, Citizen Scientists were essential partners in co-creating the VSE for our studies.

### The present studies

The present research comprises two studies based on the VSE co-created with Citizen Scientists. The VSE aims to experimentally induce social identity with a new family or friendship group (Studies 1 and 2), simulate a health crisis, and experimentally manipulate injunctive and descriptive social norms regarding protective behaviors using a vignette (Study 1) and using the VSE (Study 2). Citizen Scientists were genuine co-creators of the VSE, entitled „The field calls! “, generating implementation ideas based on the theoretical concepts developed by the research team and taking an active role in programming, testing, and iteratively refining the VSE.

Study 1 aims to test whether social identity can be experimentally induced in the VSE with a virtual in-group. Specifically, we examine whether it is possible to foster social identity with two distinct types of groups (a family and a friendship group) and compare these conditions with a low-identity control condition. For the social identity manipulation part of the VSE, farming with either a friend or family in-group against a neighboring out-group is simulated over the course of six rounds (i.e., 15-min of gaming). After the social identity manipulation (referred to as Part 1) in Study 1, we introduce a vignette describing a health crisis in the game world, the eruption of a volcano that causes a contagious disease, and mandated protective behaviors (injunctive norm) that are either followed or not by the in-group (descriptive norm). We assess social identity behaviorally and using self-report measures (description of all measures below). The main research question of Study 1 is as follows: Is it possible to induce a social identity with a virtual family or friendship group?

Study 2 aims to investigate the causal relationships between the degree of social identity with a family or friendship group, injunctive and descriptive social norms regarding protective behaviors, and the participant’s adherence to protective behaviors in a simulated health crisis. In this study, we use the same six-rounds of social identity manipulation with a family or friendship group (Part 1) and afterwards, participants play additional six rounds in the VSE with a simulated health crisis (referred to as Part 2), in which a contagious disease results from a volcano explosion and protective behaviors are mandated (injunctive norm) and either followed or not by the in-group (descriptive norm). We assess adherence to protective behaviors behaviorally, social identity behaviorally and using self-report measures, and perceived social norms using self-report measures (description of all measures below). The central research questions of Study 2 are: Does the degree of social identification affect the adherence to protective behaviors depending on the descriptive norm of the in-group? Do the effects differ when social identity is induced with a family versus friendship group?

The main hypotheses of both studies are (see [4, 5] for all preregistered hypotheses and planned analyses):

The greater the perceived strength of the in-group norm, the higher the adherence to normative in-group behaviors (H1).

The greater the perceived strength of the in-group norm and the stronger the alignment of the in-group norm with the injunctive norm, the higher the adherence to normative in-group behaviors. (H2).

The association between the in-group norms and the adherence to normative in-group behaviors is moderated by in-group type: In friendship groups, we expect a stronger association between perceived strength of the in-group norm and adherence to normative in-group behaviors than in family groups (H3).

The association between the in-group norms and the adherence to normative in-group behaviors is moderated by social identity: The stronger the social identity with the in-group, the stronger the association between the perceived strength of the in-group norm and adherence to normative in-group behaviors (H4).

### Design of studies 1 and 2

Participants are randomly assigned to one of the conditions in a between subject design with the factors social identity (family vs. friends vs. no identity control) and descriptive social norm of in-group in the vignette (Study 1) and in the VSE (Study 2) (adherence to protective behaviors: yes, no).

To exclude potential confounding effects related to time spent in the VSE, every gaming (i.e., engaging with the VSE) round lasts 15 min. Part 1 (Study 1 and 2) comprises six rounds of the computer game and Part 2 (only Study 2) comprises another six rounds. Two rounds can be played consecutively (i.e., session) followed by a break of 12 h before being able to participate in the next session (i.e., next two rounds). We introduced the break between sessions in order to space interactions with the in-group over time and thereby consolidate the social identity.

**Fig. 1:**
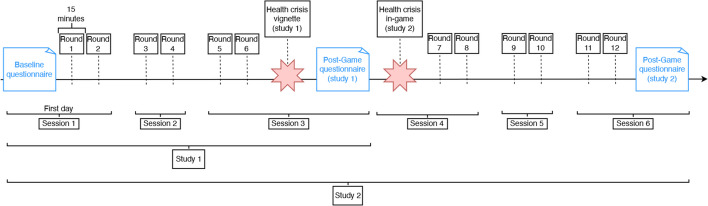
Flow of data collection for Studies 1 and 2

Figure 1 visualizes the flow of data collection for Studies 1 and 2. See the section on measures for a detailed description of measures in Baseline and Post-game questionnaire and during game rounds.

### Sample size and recruitment

We aim for a sample size of *N* = 600 for each of the studies (100 participants per condition). As we will use Bayesian statistics with no informed priors, data collection will continue until the Bayes Factors reach an informative threshold (BF10 ≥ 10) [51]. Data collection is currently ongoing. The anticipated completion date is May 1, 2026.

Inclusion criteria are being fluent in German, being older than 18 years, and having access to a personal computer or laptop and a stable internet connection. Recruitment for both studies is conducted via social media platforms (Facebook, Instagram, LinkedIn), university participant pools, fliers, and the online platform Prolific, including participants from Switzerland, Austria, and Germany. Interested people receive detailed information about the study procedures and a video explaining the participation process. After providing informed consent, participants register for the study and receive all study materials via automated emails. We run a lottery with different prizes for participants. Those who participate via Prolific receive CHF 22 for Study 1 and CHF 40 for Study 2.

### VSE „The field calls! “

The VSE developed for this study called „The field calls! “ is a farming game set in the fictional city of Mikahausen. Participants control a mouse-like avatar, a Mika, and engage in typical farming activities, e.g., planting crops. The player performs farming activities collaboratively with a virtual social group (the in-group), while there is another group of programmed avatars present that farms on a neighboring farm (out-group). The overarching storyline that is introduced in the beginning of the game (Part 1) involves an impending natural disaster: a nearby volcano has become active, and participants, together with their in-group, must earn as much money as possible through farming to relocate to a safe area. This volcanic eruption occurs in the vignette after round 6 (Study 1) and within the VSE at the beginning of round 7 (Part 2, Study 2).

**Fig. 2:**
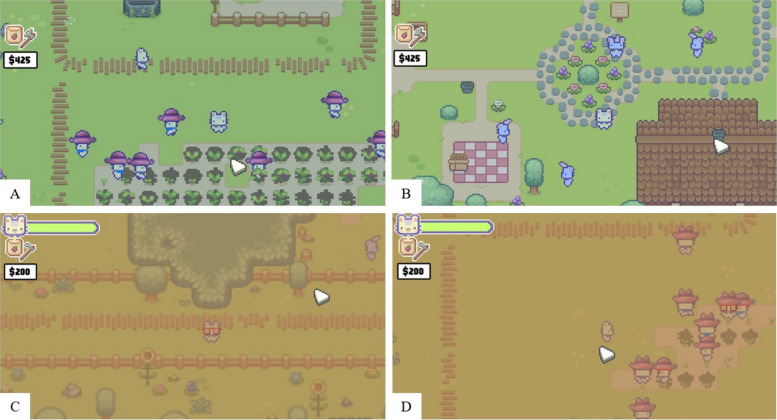
Screenshots of „The field calls! “. Note: Panel **A**: participant’s avatar in the center with in-group members on their farm; Panel **B**: participant’s avatar in the center with out-group members on the town map; Panel **C**: left participant’s avatar wearing goggles; Panel **D**: participant’s avatar on their farm with in-group members who wear goggles

Beyond farming, participants can interact with both in-group and out-group members. Interactions are symbol-based and include expressions of positive and negative affect (love, happiness, contentedness, sadness, anger). The VSE has three maps (farm, town, and forest), which participants can freely explore and switch between at any time. Because participants can choose their own actions and movement, they may miss important information or events relevant to the experimental manipulation. To address this, we integrated several timed cut scenes (i.e., short video-like sequences) to ensure that all participants receive key information [42]. Figure 2 shows several screenshots of the game.

The VSE was programmed using Pygame-ce, a Python-based library, and distributed via the Pygbag library, enabling engagement in the VSE through a web browser. A stable internet connection is required because decisions and behavioral data in the VSE are logged and transmitted to a database in real time. One can only engage in the VSE on a personal computer and laptop. In this study, Citizen Scientists co-created the VSE. While the research team set the theoretical constructs, Citizen Scientists translated them in an enjoyable and user-friendly VSE. In addition, Citizen Scientists programmed and tested the VSE. The VSE development process was conducted entirely online and was open to all people interested in participating. To characterize the Citizen Scientists and evaluate their experiences, a survey was conducted and is reported in the following section.

### Involvement of Citizen Scientists

After we specified the design of the studies and the operationalization of the theoretical constructs, we recruited Citizen Scientists via the Citizen Science Center Zurich, higher education institutions, including universities with game development programmes, and the Swiss Game Hub, a Zurich-based community of professional and hobbyist game developers. We planned several recruitment waves, as we anticipated that the interest of Citizen Scientists might decrease over time. One of the first recommendations from the Citizen Scientists was to modify an existing VSE rather than developing a new one from scratch, a strategy successfully applied in previous research (e.g., [52]). Modifying an existing VSE enabled us to produce a tailored version within a shorter timeframe. A suitable VSE for modification was identified on YouTube [53], and its source code was publicly available on GitHub [54]. Permission to adapt the code for research purposes was requested from, and granted by, the original author.

To facilitate development of the VSE, we created a dedicated Discord Server for communication and distribution of information and a GitHub repository for coding. Suggested features for the VSE, that mirror theoretical concepts, were first reviewed by us and then organized as implementation tasks using GitHub’s task list functionality. For example, drawing on SIT [33, 34], the in-group should be visually distinct from the out-group. Citizen Scientists worked out the best way to visually distinguish between these groups. Once a suggestion was approved by the research team, the corresponding programming tasks were uploaded to GitHub. Citizen Scientists played a central role in moderating the Discord server, maintaining the GitHub repository, organizing project information, and coordinating other Citizen Scientists. They also introduced and instructed new Citizen Scientists, e.g., by answering questions. The VSE coding followed a standard GitHub workflow, including commits, pull requests, code reviews, and merges. At the conclusion of the programming phase, all Citizen Scientists received a personalized certificate from the University of Zurich, formally acknowledging their co-creation of the study.

We conducted a survey among the Citizen Scientists who participated in the development of the VSE. *N* = 41 out of 63 active Citizen Scientists participated in the survey. The results are summarized in Table 1. All items used a response scale ranging from 0 to 6 that provided verbal anchors for the extremes adapted to the item (e.g., not at all – very much; never – very often).

**Table 1:** Characteristics and Self-Reported Experiences of Citizen Scientists

Characteristic	Description
Age	15–45 years; *M* = 22.87 years, *SD* = 7.84
Gender	12% female, 88% male
Occupational status	69% students; 29% employed; 2% unemployed
Country of residence	United States (22.5%), India (10%), United Kingdom (7.5%), Poland, Germany, France, Pakistan (each 5%), Bangladesh, the Netherlands, Vietnam, the Philippines, Egypt, Singapore, Indonesia, Mexico, Algeria, Bosnia and Herzegovina, Portugal, Spain, Hungary, Italy (each 2.5%)
Recruitment channels	YouTube (55%), Pygame-ce Discord server (21%), Clear Code Discord server (19%), friends or colleagues (2%), Swiss Game Hub (2%)
Overall participation experience	*M* = 6.38, *SD* = 0.8
Prior participation in Citizen Science projects	*M* = 2.24, *SD* = 1.48
Motivation for participation	
Contributing to scientific research	*M* = 5.79, *SD* = 1.64
Entertainment	*M* = 5.69, *SD* = 1.73
General interest in research	*M* = 5.67, *SD* = 1.49
Perceived learning through participation	
Coordinating with others	*M* = 5.63, *SD* = 1.46
GitHub/Git	*M* = 4.89, *SD* = 1.91
game mechanics	*M* = 4.89, *SD* = 1.86
Increased interest through participation	
GitHub/Git	*M* = 4.97, *SD* = 1.97
Pygame(-ce)	*M* = 4.45, *SD* = 2.19
Python	*M* = 4.24, *SD* = 2.10

### Manipulations

#### Identification with the avatar

To enhance identification with the avatar, participants personalize their avatar by assigning it a name (max. 16-characters) before the game starts. This personalized name is then consistently used in the VSE.

#### Social identity manipulation

We implemented several elements to promote participants’ social identity with their designated in-group. Note that we aim to induce a social identity as strongly as possible using a combination of different measures to do so; it is not a goal of the current study to test the effectiveness of any specific technique. The techniques are summarized in Table 2.

**Table 2:** Overview of social identity manipulation techniques implemented in the VSE

Manipulation	Implementation in the VSE	Reference
Group goal and common fate	We use the narrative that the player and their in-group collaborate on the common goal of farming together to earn enough money to escape from a farm threatened by a volcanic eruption	[47, 55, 56]
Minimal group	We consistently frame the in-group as a cohesive entity and refer to it using a collective name	[57–60]
Visual differentiation	In-group members share distinct visual features that clearly differentiate them from the out-group	[58, 60–62]
Group hierarchy and symbols	Exclusive symbolic items are provided through rituals. These items are acquired progressively, reflecting increasing group integration and granting privileges over time	[61]
Intergroup competition	A competitive mini-game of chasing cows into the barn against the out-group aims to reinforce social identity. Additionally, participants receive periodic updates comparing their in-group’s earnings with those of the out-group, with relative performance alternating across rounds	[10, 63]
Collective decisionmaking	In group gatherings and discussions, participants decide jointly with their in-group on activities such as which items to sell at the market	[60]
Repeated interaction	Social identity is maintained and strengthened over time by repeated engagement with the social group across three sessions	[15, 64]

#### Social norms manipulation

In Study 1, we manipulate social norms using a vignette presented after the final game session mimicking Part 2 of the VSE used in Study 2. Participants are introduced to a fictional health crisis scenario in the game world in which a volcanic eruption releases toxic ash, leading to a contagious disease affecting the eyes and skin. The government mandates two protective behaviors (i.e., injunctive norms): (i) wearing special goggles to protect the eyes, and (ii) taking daily baths in a special liquid to protect the skin. The descriptive social norms of the in-group and the out-group regarding these behaviors are described as follows, depending on experimental condition: (1) most in-group members consistently adhere to the protective behaviors, or (2) most in-group members do not adhere to the protective behaviors. In both conditions, 50% of out-group members adhere to the protective behaviors.

In Study 2, the health crisis and the social norms manipulation are directly integrated into the VSE. A volcanic eruption introduces the health crisis in the beginning of round 7 (beginning of Part 2), and the government in the VSE mandates the two protective behaviors (wearing goggles and taking the bath) through an on-screen message displayed shortly after the eruption. As in the vignette in Study 1, the in-group either predominantly adheres to the protective behaviors or only marginally adheres, while adherence within the out-group is 50% throughout. Different to the vignette in Study 1, the VSE directly shows the adherence behaviors of both in-group and out-group members and the health consequences of non-adherence are visually depicted (e.g., sick Mikas turning green or dying).

Similar to the Covid-19 pandemic, the two injunctive norms mandated by the government in the VSE mimic face mask wearing (represented by wearing goggles in the VSE) and vaccination (represented by taking the bath in the VSE). Consistent with the pandemic situation, one injunctive norm mandated by the government in the VSE is visible (wearing goggles), allowing constant visual observation of adherence, whereas the other is invisible (taking the bath), as it occurs privately within a hidden house in the VSE. Also mimicking the course of the Covid-19 pandemic, injunctive norms within the VSE become less strict over time. The in-group’s adherence behavior (i.e., the descriptive norm) remains constant across sessions, while participants retain full autonomy to decide whether to adhere to the protective behaviors at any time. Participants can access static images displaying statistics on the effectiveness of the two protective behaviors.

### Health consequences and probabilities

The probability of staying healthy is calculated every five minutes based on whether the bath was taken and the duration the goggles were worn in the previous five minutes. The probabilities to stay healthy are as follows: (a) neither measure taken: 10%; (b) only bath or only goggles: 30%; (c) both measures: 60%. Sickness occurs immediately after the probability calculation and lasts for four minutes. Sick Mikas display visible symptoms (green coloring, a sick emoji face in the thought bubbles), move at 50% of normal speed, and fail in 50% of farming tool uses. Health, which is displayed with a visual health bar, decreases and recovers fully after four minutes. In-group and out-group members follow pre-programmed probabilities with sickness differing by experimental condition (e.g., in rounds 7–9, eight non-adhering in-group members get sick in the non-adherence condition vs. two in the adherence condition). Deaths occur selectively (e.g., two in-group members in rounds 7–9 of the non-adherence condition vs. one in the adherence condition). To ensure that all participants engage in the same number of rounds and provide an equal number of behavioral and self-report measures; the participant’s avatar cannot die.

### Costs of adherence

To simulate the costs many people experienced when wearing face masks and getting vaccinated during the Covid-19 pandemic, we introduced similar costs when taking the bath and wearing goggles. The bath is only effective if taken while healthy and requires a 25-s walk from the farm. It induces mild sickness symptoms for one minute. If taken shortly before a probability calculation (e.g., at minute 9:59), the calculation is postponed by one minute to account for the bath’s protective effect. Goggles blur the view in the VSE, similar to feeling constrained when breathing using a face mask.

### Control condition

In the control condition, no social identity manipulation exists. Neither the in-group nor the out-group is presented as a cohesive entity; for instance, the term “group” is never used. No exclusive items, hierarchical structures, initiation rituals, or group discussions take place. Participants still cooperate on the common farming task, but the narrative framed their presence in Mikahausen as the result of random assignment to this location. The manipulation of social norms is identical to that in the experimental conditions.

### Behavioral measures

All measures, including behavioral and self-report, can be found on OSF [4, 5].

#### Adherence to protective behaviors

The adherence measures are logged in Study 2, including: 1) whether and when participants equipped or removed the goggles, 2) taking the bath, including attempts to take the bath while sick (despite the bath having no effect in this state).

#### Social identity

All behavioral indicators of social identity are theoretically embedded in Social identity theory [34], Self-categorization theory [11], and Entitativity theory [61] and listed in Table 3.

**Table 3:** Overview of social identity indicators measured in the VSE

Indicator	Operationalization in the VSE	Reference
Allocation of resources	Once every 15 min, participants distribute a farming resource (e.g., gloves) between their in-group and the out-group	[57, 65]
Social interactions	We record both in-group and out-group interactions, including their frequency and type (positive or negative)	[66]
Farming engagement and proximity to in-group	We measure engagement in the shared group goal (i.e., earning money through farming) by logging the frequency and type of farming activities, which also captures proximity to in-group members	[55, 66]
Group-switching behavior	In Part 2 (only Study 2), participants can permanently leave their designated in-group to join the out-group and farm together. This choice persists across subsequent rounds	[10]
Engaging in intergroup comparison through competition	We track how often participants play the competitive mini-game against the out-group	[10, 11]

### Self-report measures questionnaire

In both studies, participants complete a Baseline questionnaire before the first game session assessing basic demographic characteristics (age, gender, education, income, occupation, nationality, civil status) as well as subjective health, life satisfaction, loneliness, chronic illness, trust, and perceived threat to freedom. We also ask for the level of social identification with their actual family and friends. In addition, we assess the following control variables: Attitudes towards Covid-19 protective behaviors and experiences with computer games. A detailed description of all Baseline questionnaire items can be found on OSF [4, 5].

### Assessments in each game round

In each game round of Study 1 and 2, participants rate their level of social identification with the in-group and the out-group. We also assess emotional well-being (valence, arousal) and perceived control. In Part 2 of Study 2, participants additionally rate the perceived risk from the disease, response efficacy, self-efficacy and perceived social norms of the in-group regarding the protective behaviors.

A detailed description of all assessments in each game round can be found on OSF [4, 5].

### Post-game questionnaire

The questionnaire after the last game session in Study 1 and 2 includes assessments of social identification with the in-group and the out-group. In addition, we assess identification with the player’s avatar. In Study 1, after the vignette, we also assess intention to wear the goggles and take the protective bath, perceived risk from the disease, response efficacy, self-efficacy and perceived social norms of the in-group regarding the protective behaviors. In Study 2 for both in-group and out-group, we additionally assess moralization, perceived moralization, moral condemnation, cognitive/affective empathy, social control, punishment, and social support. A detailed description of all Post-game questionnaire items can be found on OSF [4, 5].

### Statistical analysis

All planned statistical analyses were preregistered and can be found on OSF [4, 5]. Here, we will only list the central analyses pertaining to main hypotheses of Study 2 in Table 4. We will use Bayesian multilevel models for all tests and include age [67, 68], gender [67–69], belonging to a risk group [67, 70–72], marital status [68, 69], and income and education [67–69, 73] as control variables in sensitivity analyses.

**Table 4:** Overview of planned statistical analyses to test Hypotheses 1–4

Hypothesis	Model specification
H1	adherence ∼ perceived norm strength
H2	adherence ∼ perceived norm strength + norm alignment + perceived norm strength × norm alignment
H3	adherence ∼ perceived norm strength + group type + perceived norm strength × group type + norm alignment
H4	adherence ∼ perceived norm strength + social identity + perceived norm strength × social identity + norm alignment

## Discussion

Adherence to protective behaviors is critical for reducing infectious disease spread [1–3], yet the Covid-19 pandemic demonstrated substantial variability in adherence [74]. We developed a new paradigm using a VSE that allows to test theoretically derived causal hypotheses of factors impacting the adherence to protective behaviors. More specifically, we designed and co-created a computer game together with Citizen Scientists that experimentally manipulates social identity with different social groups as well as social norms during a pandemic-like health crisis. Our new paradigm has the potential to contribute to experimental research around social identity, as it can be used to manipulate social identity and other variables, such as social norms, and implement various simulations. In addition, we show that VSEs can be successfully co-created with Citizen Scientists.

The Citizen Science co-creation of the VSE has several strengths. The input of Citizen Scientists led to the generation of new ideas for the content of the VSE, which the research team alone would not have been able to produce. This made the resulting VSE more appealing and relevant for study participants than if the VSE had been developed by a programming company. In addition, Citizen Scientists were able to apply their programming skills to a meaningful research project and experiencing several learning gains in the process, such as learning how to coordinate with others.

The development of the new paradigm also posed practical challenges. Engagement of Citizen Scientists largely occurred during evening or night hours, increasing coordination demands, which were further amplified by the size and complexity of the VSE. Some Citizen Scientists proposed game features beyond the study aims, creating tension between their involvement on the one hand and the methodological requirements of the experimental design on the other. We addressed these tensions by providing a detailed development timetable with clear milestones and explicit prioritization of study relevant features. The engagement of the Citizen Scientists declined toward the end of the development of the VSE. To counter declining engagement, we posted weekly progress updates on Discord with specific coding tasks and encouragements. Completion ultimately required intensive in-person contributions from two Citizen Scientists who resolved remaining technical issues. Prior VSE studies either relied on fully in-house programming, reported as highly time intensive [42, 52], or did not specify implementation responsibilities [45–47]. In contrast, our Citizen Science approach represents a feasible alternative and resulted in a visually engaging, enjoyable, and intuitive VSE. Regarding the social identity manipulation, we faced some conceptual challenges. There exists currently no systematic evidence comparing the effectiveness of different methods to induce social identity with a virtual group. For instance, it is as of yet not known if the visual differentiation between in-group and out-group members (e.g., in-group members appearing silver and out-group members appearing purple) is more effective than assigning a specific group name (e.g., „The Redhats “). For this reason, we opted for a kitchen-sink approach and integrated as many methods to induce social identity with the virtual group as was possible within the game context. Regarding the manipulation of social norms, we do not know if participants are fully aware of the adherence or non-adherence behavior of their in-group members, since we manipulate descriptive social norms regarding protective behaviors visually (e.g., in-group members do or do not wear the goggles; we did not make the in-group social norm explicit via text). To check how participants perceive their in-group’s social norm regarding protective behaviors, and in order to compare the perceived social norm with the manipulation, we assess perceived social norms in every round.

We anticipate participant drop-out due to technical issues such as unstable internet connections, study duration, daily gaming requirements, or a lack of interest in gaming. To reduce disengagement and drop-out, we designed the VSE to be engaging and easily accessible, incorporating input from experienced Citizen Scientists, written gameplay manuals, and an instructional video explaining participation procedures. We further anticipate a selection bias toward participants with an affinity for computer games. We attempt to mitigate this selectivity by explicitly encouraging participants without gaming experience. In the recruitment process, we emphasize low barriers to participation, including no software installation and automated transitions between questionnaires and the VSE. Nevertheless, people without access to a personal computer, laptop, or mobile internet are excluded by design. However, such selectivity applies to all online studies, for which, in general, very few – if any – differences to other convenience samples have been identified [75, 76]. Regarding the social identity manipulation, we anticipate little differences between the family and the friendship group since, in both cases, participants are assigned to their respective group. However, unlike in our VSE, real families cannot be chosen, whereas friendship groups can. Consequently, the friendship condition in our VSE does not accurately mimic the development of a real friendship group.

## Conclusion

Understanding why people do or do not adopt protective behaviors remains central to managing future infectious disease threats. The paradigm introduced here enables controlled experimental investigation of social identity and social norms in a VSE that approximates real-world social processes more closely than traditional approaches. At the same time, the Citizen Science co-creation approach proved highly successful. We view this work as a foundation for future research to systematically manipulate social identity and social norms to study their causal relationship, and assess behavior directly in virtual social environments. A wide range of simulations and respective social norms can be simulated within VSEs, which makes it a practical tool for various research questions regarding the interplay of social norms and, for example, social identity.

## Data Availability

Data on Citizen Scientists presented in this study protocol will be available on OSF: 10.17605/OSF.IO/C2UDE.

## Abbreviations

VSE: Virtual social environment
SIT: Social identity theory
FTNC: Focus theory of normative conduct
NAM: Norm activation model
SCT: Self-categorization theory
PPSR: Public Participation in Scientific Research
